# The association between dietary patterns and metabolic syndrome among Iranian adults, a cross-sectional population-based study (findings from Bandare-Kong non-communicable disease cohort study)

**DOI:** 10.1186/s12902-024-01584-7

**Published:** 2024-04-30

**Authors:** Masoumeh Kheirandish, Farideh Dastsouz, Abnoos Azarbad, Mohammad Ali Mohsenpour, Gholamali Javdan, Farkhondeh Razmpour, Seyed Hossein Davoodi, Nahid Ramezani-Jolfaie, Mohammad Mohammadi

**Affiliations:** 1 Endocrinology and Metabolism Research Center, Hormozgan University of Medical Sciences, Bandar Abbas, Iran; 2 Food Health Research Center, Hormozgan University of Medical Sciences, Bandar Abbas, Iran; 3 Department of Clinical Nutrition, School of Nutrition and Food Sciences, Shiraz University of Medical Sciences, Shiraz, Iran; 4 Student Research Committee, Shiraz University of Medical Sciences, Shiraz, Iran; 5 Department of Community Medicine, School of Medicine, Hormozgan University of Medical Sciences, Bandar Abbas, Bandar Abbas, Iran; 6 Department of Clinical Nutrition, School of Nutritional Sciences and Food Technology, Shahid Beheshti University of Medical Sciences, Tehran, Iran

**Keywords:** Dietary patterns, Metabolic syndrome, Blood glucose, Waist circumference, Blood pressure, Prospective Epidemiological Research Studies in Iran (PERSIAN)

## Abstract

**Background:**

Metabolic syndrome is a cluster of metabolic disorders increasing the risk of cardiovascular disease and diabetes. Dietary patterns are supposed to be important and controllable factors in developing metabolic syndrome. The purpose of this study was to investigate the association of dietary patterns with metabolic syndrome and its components.

**Subjects/Methods:**

Cross-sectional data were extracted from the Bandare-Kong cohort study conducted on 4063 people aged 35 to 70. Dietary patterns were extracted using principal component analysis based on thirty-eight pre-defined food groups. Multivariable logistic regression was conducted to investigate the association between metabolic syndrome and its components with quintiles of dietary patterns in crude and adjusted models.

**Results:**

Three major dietary patterns were identified (healthy, western, and traditional) in the final analysis of 2823 eligible individuals. After adjusting for covariates, the odds of metabolic syndrome were significantly decreased by 46% in subjects with the highest adherence to the healthy dietary pattern compared to those with the lowest adherence quintile. Results from fully adjusted models on individual metabolic syndrome components showed an inverse association between higher adherence to the healthy dietary pattern and the odds of increased blood glucose, high waist circumference, and elevated blood pressure. However, in fully adjusted models, no significant association was observed between the western and traditional dietary patterns with odds of metabolic syndrome and its components.

**Conclusions:**

Adherence to a healthy dietary pattern containing high amounts of fruits, vegetables, nuts, low-fat dairy products, and legumes, could be recommended to prevent and control metabolic syndrome.

**Supplementary Information:**

The online version contains supplementary material available at 10.1186/s12902-024-01584-7.

## Introduction

Metabolic syndrome is a public health concern that affects approximately a quarter of all adults worldwide. It is not a disease but a cluster of metabolic disorders, increasing the risk of cardiovascular disease and stroke by up to two-fold and the risk of diabetes by up to five-fold [1]. According to NCEP-ATP III criteria, metabolic syndrome is defined as the presence of three of the following five indicators including elevated waist circumference (> 102 cm in men or > 88 cm in women), elevated blood pressure (systolic ≥ 130 and/or diastolic ≥ 85 mm Hg), elevated triglycerides (≥ 150 mg/dL), reduced high-density lipoprotein cholesterol (< 40 mg/dL in males; <50 mg/dL in females), and elevated fasting blood glucose (≥ 100 mg/dl) [2].

The importance of dietary regimens and specific nutrients in the pathophysiology of metabolic syndrome is well acknowledged [3]. Dietary habits are one of the most important lifestyle-related risk factors in this disorder and there is a growing interest in the study of dietary patterns as a whole instead of individual dietary components associated with metabolic syndrome which can be an effective step toward eliminating and preventing the condition [4–6]. Among the research in this field, we can mention a systematic review and meta-analysis of observational studies that showed adherence to a “healthy” dietary pattern (high consumption of fruit, vegetables, whole grains, poultry, fish, nuts, legumes, and low-fat dairy products) was associated with a reduced risk of metabolic syndrome, while a “meat/western” dietary pattern (rich in red and processed meat, eggs, refined grains, and sweets) was associated with an increased risk [7]. In a nutshell, recent evidence supports the protective effects of applying healthy food-based dietary patterns due to the sum of small dietary changes rather than the restriction of individual nutrients or calories [8].

Regional diversity in factors like dietary habits and disease prevalence provides a good opportunity to investigate such associations. As considering the geographic locations of the Bandare-Kong Non-Communicable Diseases (BKNCD) cohort study in a southern coastline of Iran with specific socio-demographic and lifestyle features in addition to the high prevalence of metabolic syndrome in this region (34%) [9], give us this view of examine the dominant dietary patterns and then analyzed whether these dietary patterns are associated with metabolic syndrome. It is important to note that no major studies have yet been conducted to determine dietary patterns in the southern of Iran, especially in coastal areas that are important in terms of seafood consumption. Consequently, this study was done to determine prevalent dietary patterns in the population of the BKNCD cohort study as besides aimed at investigating the relationship between the identified patterns and metabolic syndrome and its constituents.

## Subjects and methods

### Study population and data collection

This cross-sectional survey was performed using the baseline data of the BKNCD cohort study, conducted as part of a prospective epidemiological research study in Iran (PERSIAN) in Bandare-Kong, a harbor city, in the southernmost point of Iran. Participant recruitment was undertaken between October 2016 and November 2018. The protocol for the BKNCD cohort study is fully described in separate articles [10, 11]. The study involved 4063 adults between the ages of 35 and 70, and the statistical analysis was finally run for 2823 eligible individuals. Pregnant women, people with chronic diseases such as cardiovascular disease, diabetes, and cancer, as well as people with energy intakes less than 800 kcal and more than 4200 kcal, were excluded from the study. Demographic and socioeconomic status (age, gender, and employment status), smoking habits, and dietary intakes were collected in a face-to-face setting by trained interviewers.

### Anthropometric measurements

Participants’ weight was measured with minimal clothing, using a mechanical scale (SECA, model 755, Germany) with an accuracy of 0.1 kg, and their height was measured without shoes using a non-stretch tape measure and with an accuracy of 0.5 cm. Body mass index (BMI) was obtained by dividing weight in kilograms by height in meters squared. Waist circumference (WC) was measured at the end of several consecutive natural breaths at a level parallel to the midpoint between the top of the iliac crest and the bottom margin of the last palpable rib, using a stretch-resistant tape. Hip circumference (HC) was also measured at the highest point of the buttocks using a flexible tapeline.

### Physical activity

Physical activity was assessed using a validated questionnaire that recorded the daily activities reported by participants in the past year. The metabolic equivalent (MET) of each activity was extracted using a compendium of physical activities and was calculated over 24 h based on reported activities. The weekly average of physical activities, including leisure time, work, and sports activities, were collected as MET-min/week [12, 13].

### Smoking definition

Smoking status of participants was categorized into smokers and non-smokers based on self-reported data by answering the question whether have smoked at least 100 cigarettes in their lifetime (yes/no) [14].

### Dietary pattern assessment

The food intakes were obtained using a modified semi-quantitative food frequency questionnaire (FFQ) containing 132 food items [15]. The consumption of each food item per year was recorded according to its consumption pattern in the day, week, month, or year. In the next step, food intakes were converted into g/day for data analysis. The amount of energy and nutrients were received from the Nutritionist IV software. All data were entered into SPSS 25 software for analysis. In order to reduce complexity, 132 food items were classified into 38 food groups based on nutrient similarity and culinary usage. Then, dietary patterns were obtained using principal component analysis with varimax rotation based on 38 food groups. Dietary patterns were identified according to various factors, such as eigenvalues > 1, rotational factor load > 0.3, and clear inflection in the scree plot.

### Blood pressure measurement and laboratory investigation

Blood pressure (BP) was measured twice after 5 min of rest using a standard mercury sphygmomanometer (Riester, CE 0124, Germany) while the person was sitting with the arm at the level of the heart, and the mean value was recorded. Participants were referred to the laboratory for the collection of blood samples. Enzymatic methods were used to measure fasting blood glucose, total cholesterol, triglycerides, low-density lipoprotein cholesterol (LDL-C), and high-density lipoprotein cholesterol (HDL-C) following a 12-hour fast.

### Statistical analysis

All analyses were performed using SPSS (version 25) software. The quantitative variables were represented using means ± standard deviations (SD), and categorical variables were expressed using percentages. Analysis of variance and Chi-square tests were used to compare quantitative and qualitative variables across the quintiles of adherence to dietary patterns. A comparison of age, sex, and energy-adjusted micro- and macro-nutrients, as well as food groups’ intake according to quintiles of major dietary patterns, was performed using the ANCOVA test. Multivariable logistic regression was used to investigate the odds ratio (OR) with a 95% confidence interval (CI) of metabolic syndrome and its components among quintiles of dietary patterns in crude and adjusted models controlling for age, sex, energy intake, BMI, physical activity, education level, marital status, and smoking.

## Results

The principal component analysis, based on 38 pre-defined food groups, identified three major dietary patterns explaining 21% of total variances as follows: healthy dietary pattern (high in vegetables, fruits, yellow vegetables, leafy green vegetables, cruciferous vegetables, nuts, tomatoes, dried fruits, olives, and dairy products), western dietary pattern (high in soft drinks, sweets and desserts, condiments, pizza, red meats, snacks, poultry, refined grains, mayonnaise, canned fish, eggs, processed meats, and high-fat dairy products) and traditional dietary pattern which was high in sugars, tea, salt, potatoes, hydrogenated fats, and coffee. The food groups and their relevant factor loadings for each posterior dietary pattern are presented in Supplementary Tables 1 and 2, respectively.

Among the 4063 participants of the BKNCD cohort study, 2823 (40.3% males and 59.7% females, aged 35 and 70) were included in the final analysis. Table 1 summarizes the general characteristics of participants across quintiles of identified dietary patterns. Those with the highest adherence to the healthy dietary pattern had higher weight, BMI, hip circumference (*P* < 0.001, for all), and waist circumference (*P* = 0.006). Individuals with the highest adherence to the western dietary pattern had higher weight (*P* < 0.001) but lower waist circumferences (*P* = 0.020) compared with the lowest. There were differences in age, level of physical activity, and marital status among the lowest and highest quintiles of the western and traditional dietary patterns (*P* < 0.05).

**Table 1:** General characteristics of study participants according to quintiles of adherence to major dietary patterns^1^

	Healthy Dietary Pattern			Western Dietary Pattern			Traditional Dietary Pattern		
	Q1	Q5	*P*-value^2^	Q1	Q5	*P*-value^2^	Q1	Q5	*P*-value^2^
Subjects (*n*)	498	525		505	519		524	506	
Sex (female) (%)	19.6	18.6	0.077	78.6	32.4	< 0.001	59.5	52.2	0.008
Age (year)	47.48 ± 9.64	46.76 ± 8.42	0.379	50.87 ± 9.99	43.57 ± 7.27	< 0.001	44.21 ± 7.97	47.59 ± 8.73	< 0.001
Weight (kg)	67.43 ± 14.37	73.38 ± 14.73	< 0.001	67.76 ± 14.26	74.00 ± 14.32	< 0.001	70.77 ± 15.27	70.72 ± 14.41	0.868
Body mass index (kg/m^2^)	25.93 ± 5.21	27.27 ± 4.84	< 0.001	26.85 ± 5.36	26.57 ± 4.70	0.432	26.60 ± 5.07	26.55 ± 5.06	0.790
Waist circumference (cm)	91.52 ± 12.55	94.01 ± 11.43	0.006	93.73 ± 12.29	92.11 ± 11.30	0.020	92.57 ± 12.23	92.16 ± 11.65	0.386
Hip circumference (cm)	98.13 ± 9.29	101.16 ± 9.59	< 0.001	100.36 ± 10.35	100.16 ± 8.95	0.513	100.27 ± 9.50	99.67 ± 9.93	0.591
Physical activity (MET-min/week)	39.94 ± 5.82	40.90 ± 6.19	0.128	40.15 ± 5.32	41.05 ± 6.60	0.001	40.28 ± 5.42	41.34 ± 7.01	0.005
Marital status (%)									
Single	3.0	3.0	0.012	4.0	2.3	< 0.001	4.6	2.0	0.060
Married	87.3	90.1		82.0	94.8		89.7	91.1	
Widowed or divorced	9.6	6.9		14.1	2.9		5.7	6.9	
Education									
illiterate	10.3	4.8	< 0.001	10.4	5.3	< 0.001	5.1	8.6	< 0.001
Secondary school	7	8.5		5.3	9.5		8.8	7.6	
Diploma	1.5	3.4		2.3	2.8		3.4	2.1	
Bachelor’s degree	0.5	3		1.4	2.2		2.5	1.2	
Master’s degree and higher	0.1	0.8		0.3	0.5		0.6	0.2	
Smoking status (%)									
Smoker	86.5	87.2	0.224	92.7	22.5	< 0.001	92.2	76.8	< 0.001
Non-smoker	13.1	12.8		7.3	77.3		7.8	23.2	

^1^ Values are reported as mean ± standard deviation (SD) otherwise indicated

^2^ The quantitative and qualitative variables were compared across quintiles of adherence to dietary patterns using the analysis of variance and Chi-square tests, respectively

Table 2 shows participants’ dietary intake in quintiles of the major dietary patterns. Subjects in the fifth quintile of the healthy dietary pattern had significantly higher intakes of energy, fruits, vegetables, legumes, dairy, nut, fiber, magnesium, calcium, vitamin C, B6, and B9 and lower intakes of processed meat and refined grains compared with those in the first quintile. Also, with more adherence to the western dietary pattern, fruits, nuts, dairy products, fiber, trans fatty acids, calcium, vitamin C, B6, and B9 intakes tended to decrease and the intakes of energy, processed meat, refined grain, and saturated fat were increased.

**Table 2:** Comparison of age, sex, and energy-adjusted micro- and macro-nutrients, as well as food groups’ intake according to quintiles of major dietary patterns^1^

	Healthy Dietary Pattern			Western Dietary Pattern			Traditional Dietary Pattern		
	Q_1_	Q_5_	*P*-value	Q_1_	Q_5_	*P*-value	Q_1_	Q_5_	*P*-value
Total energy (Kcal/day)^2^	2269.04 ± 589.62	3171.99 ± 589.47	< 0.001	2128.95 ± 559.25	3389.75 ± 470.14	< 0.001	2561.17 ± 665.22	3034.13 ± 642.85	< 0.001
*Nutrients*^*3*^									
Total fat (g/day)	73.55 ± 0.87	76.00 ± 0.83	0.016	66.57 ± 0.89	87.63 ± 0.89	< 0.001	75.44 ± 0.80	75.44 ± 0.82	0.016
Saturated fat (g/day)	27.27 ± 0.44	27.10 ± 0.42	0.645	23.87 ± 0.46	32.31 ± 0.46	< 0.001	27.09 ± 0.41	28.06 ± 0.41	0.028
Mono-unsaturated fat (g/day)	24.03 ± 0.35	25.54 ± 0.34	0.001	21.62 ± 0.37	29.59 ± 0.37	< 0.001	25.21 ± 0.33	25.05 ± 0.33	0.001
Poly-unsaturated fat (g/day)	11.59 ± 0.14	12.69 ± 0.13	< 0.001	11.12 ± 0.14	13.73 ± 0.14	< 0.001	11.71 ± 0.13	12.16 ± 0.33	0.033
Trans fatty acid	0.36 ± 0.00	0.24 ± 0.00	< 0.001	0.15 ± 0.08	0.51 ± 0.25	< 0.001	0.25 ± 0.19	0.35 ± 0.22	0.001
Polyunsaturated omega-3 fat	1.06 ± 0.02	1.10 ± 0.02	0.079	1.06 ± 0.02	1.16 ± 0.02	0.012	1.03 ± 0.20	1.11 ± 0.02	0.030
Total protein (g/day)	85.62 ± 0.58	86.45 ± 0.56	0.545	80.95 ± 0.62	90.24 ± 0.61	< 0.001	87.65 ± 0.54	83.67 ± 0.55	< 0.001
Total carbohydrate (g/day)	444.56 ± 3.39	431.33 ± 3.25	0.041	441.29 ± 3.67	427.33 ± 3.65	0.018	424.22 ± 3.13	445.50 ± 3.10	< 0.001
Simple sugar (g/day)	125.00 ± 2.10	165.06 ± 2.01	< 0.001	156.51 ± 2.34	134.98 ± 2.33	< 0.001	131.59 ± 1.98	161.18 ± 2.01	< 0.001
Total fiber (g/day)	21.12 ± 0.25	35.43 ± 0.24	< 0.001	31.97 ± 0.33	23.18 ± 0.33	< 0.001	28.37 ± 0.29	25.85 ± 0.30	< 0.001
Vitamin C (µm /d)	81.96 ± 2.40	203.47 ± 2.29	< 0.001	158.85 ± 3.16	122.37 ± 3.15	< 0.001	141.99 ± 2.74	125.33 ± 2.79	0.001
Vitamin E (mg /d)	7.72 ± 0.11	9.44 ± 0.11	< 0.001	8.51 ± 0.12	8.66 ± 0.12	< 0.001	8.00 ± 0.10	8.46 ± 0.11	0.012
Thiamine (mg/d)	0.81 ± 0.01	1.25 ± 0.01	< 0.001	1.02 ± 0.01	1.05 ± 0.01	< 0.001	1.07 ± 0.01	0.94 ± 0.01	< 0.001
Riboflavin (µm/d)	1.26 ± 0.38	1.90 ± 0.51	< 0.001	1.12 ± 0.47	1.84 ± 0.49	0.001	1.39 ± 0.52	1.58 ± 0.47	0.005
Niacin (mg/d)	19.98 ± 0.58	22.57 ± 0.55	0.003	18.53 ± 0.62	23.91 ± 0.62	< 0.001	18.74 ± 0.53	23.60 ± 0.54	< 0.001
Vitamin B6 (mg/d)	1.40 ± 0.01	2.01 ± 0.01	< 0.001	1.74 ± 0.01	1.63 ± 0.01	< 0.001	1.69 ± 0.01	1.62 ± 0.01	0.003
Folic Acid (µg/d)	275.16 ± 5.03	514.25 ± 4.81	< 0.001	423.69 ± 6.51	347.80 ± 6.49	< 0.001	389.17 ± 5.66	370.10 ± 5.75	0.159
Vitamin B12 (µg/d)	4.34 ± 0.15	5.50 ± 0.14	< 0.001	4.00 ± 0.16	6.08 ± 0.16	< 0.001	5.49 ± 014	4.41 ± 0.14	< 0.001
Magnesium (mg/day)	341.25 ± 7.67	442.31 ± 7.34	< 0.001	386.57 ± 8.44	378.49 ± 28.40	0.312	350.05 ± 7.17	428.24 ± 7.29	< 0.001
Calcium (mg/day)	556.08 ± 10.89	909.36 ± 10.42	< 0.001	767.25 ± 12.93	671.27 ± 12.87	< 0.001	739.17 ± 11.414	693.11 ± 11.32	0.075
Iron (mg/day)	15.74 ± 0.13	17.17 ± 0.12	< 0.001	16.24 ± 0.14	16.14 ± 0.14	0.063	16.23 ± 0.12	16.16 ± 0.12	0.503
Zinc (mg/day)	7.50 ± 0.06	9.10 ± 0.06	< 0.001	7.71 ± 0.07	8.82 ± 0.07	< 0.001	8.49 ± 0.06	8.02 ± 0.06	< 0.001
*Food groups*^*3*^									
Whole grains (g/day)	30.36 ± 1.54	42.64 ± 1.51	< 0.001	28.12 ± 1.67	42.34 ± 1.070	< 0.001	42.82 ± 1.46	29.53 ± 1.46	< 0.001
Refined grains (g/day)	480.74 ± 172.35	454.43 ± 179.75	< 0.001	356.28 ± 134.73	572.03 ± 171.91	< 0.001	483.39 ± 176.09	491.87 ± 165.04	< 0.001
dairy products (g/day)	102.44 ± 5.46	214.79 ± 5.38	< 0.001	195.75 ± 6.08	110.64 ± 6.17	< 0.001	214.79 ± 5.38	190.35 ± 124.96	< 0.001
Nuts (g/day)	2.93 ± 0.25	9.63 ± 0.24	< 0.001	7.25 ± 0.29	4.27 ± 0.29	< 0.001	5.06 ± 6.19	6.22 ± 7.88	0.324
Legumes (g/day)	54.06 ± 1.93	67.19 ± 1.90	< 0.001	61.27 ± 2.12	62.56 ± 2.15	0.317	64.78 ± 1.85	62.63 ± 1.85	0.074
Red meats (g/day)	11.03 ± 0.71	17.50 ± 0.70	< 0.001	9.83 ± 0.75	17.50 ± 0.70	< 0.001	22.02 ± 0.66	12.21 ± 0.66	< 0.001
Processed meats (g/day)	1.45 ± 0.13	0.33 ± 0.13	< 0.001	0.16 ± 0.73	2.64 ± 5.67	< 0.001	0.80 ± 2.92	1.12 ± 3.12	0.721
Fruits (g/day)	211.65 ± 7.65	568.98 ± 7.54	< 0.001	458.77 ± 9.60	290.57 ± 9.73	< 0.001	450.66 ± 308.54	308.54 ± 0.87	< 0.001
Vegetables (g/day)	115.10 ± 3.74	272.50 ± 3.69	< 0.001	251.03 ± 4.40	189.68 ± 125.350	0.702	172.58 ± 4.09	189.45 ± 4.09	0.027

^1^ Values are reported as mean ± standard error (SE)

^2^ Adjusted for age and sex

^3^ Adjusted for age, sex, and total energy

Table 3 shows the odds ratio of abnormal levels of metabolic syndrome components according to quintiles of dietary patterns. The fully adjusted model indicated decreased odds ratio of high blood glucose in those with the highest adherence to the healthy dietary pattern compared with those with the lowest adherence quintile (OR = 0.56, 95% CI: 0.37, 0.86, *P* = 0.011). Also, an inverse association between the adherence to the healthy dietary pattern and the odds ratio of increased waist circumference (OR = 0.28, 95% CI: 0.14, 0.56, *P* = 0.001) and elevated blood pressure (OR = 0.51, 95% CI: 0.27, 0.93, *P* = 0.008). However, higher adherence to this healthy dietary pattern was not associated with the odds ratio of high TG and low HDL levels. The metabolic syndrome components were not significantly different across quintiles of the western and traditional dietary patterns.

**Table 3:** The odds ratio of abnormal levels of metabolic syndrome components^1^ according to quintiles of dietary patterns in multivariable-adjusted model^2^

	High glucose	High triglyceride	Low HDL	High WC	High blood pressure
	Model 2^3^	Model 2^3^	Model 2^3^	Model 2^3^	Model 2^3^
Healthy Dietary Pattern					
Q1	1	1	1	1	1
Q2	0.81 (0.57, 1.16)	0.81 (0.55, 1.21)	0.74 (0.54, 1.01)	0.54 (0.31, 0.96)	0.94 (0.58, 1.51)
Q3	0.65 (0.45, 0.95)	0.94 (0.63, 1.39)	0.88 (0.64, 1.22)	0.57 (0.31, 1.03)	0.81 (0.49, 1.34)
Q4	0.72 (0.49, 1.06)	1.24 (0.83, 1.85)	0.87 (0.62, 1.22)	0.48 (0.26, 0.90)	0.58 (0.33, 0.99)
Q5	0.56 (0.37, 0.86)	1.04 (0.67, 1.62)	0.73 (0.51, 1.05)	0.28 (0.14, 0.56)	0.51 (0.27, 0.93)
*P-value*	0.011	0.295	0.306	0.001	0.008
Western Dietary Pattern					
Q1	1	1	1	1	1
Q2	1.39 (1.00, 1.93)	1.04 (0.74, 1.46)	1.06 (0.80, 1.41)	1.53 (0.92, 2.53)	1.21 (0.77, 1.90)
Q3	1.12 (0.78, 1.61)	0.93 (0.64, 1.36)	0.89 (0.65, 1.21)	0.95 (0.55, 1.63)	1.14 (0.68, 1.88)
Q4	1.31 (0.86, 2.00)	0.79 (0.51, 1.24)	1.06 (0.74, 1.51)	1.41 (0.76, 1.61)	1.88 (1.06, 3.35)
Q5	1.56 (0.94, 2.61)	0.81 (0.47, 1.40)	1.22 (0.79, 1.90)	1.31 (0.58, 2.91)	1.52 (0.72, 3.19)
*P-value*	0.189	0.270	0.617	0.636	0.093
Traditional Dietary pattern					
Q1	1	1	1	1	1
Q2	1.23 (0.87, 1.75)	0.97 (0.66, 1.42)	0.98 (0.72,1.32)	1.07 (0.63, 1.81)	1.03 (0.62, 1.71)
Q3	0.95 (0.66, 1.36)	1.11 (0.76, 1.62)	1.04 (0.76, 1.41)	0.97 (0.57, 1.66)	1.02 (0.62, 1.70)
Q4	0.70 (0.48, 1.03)	0.84 (0.56, 1.25)	1.22 (0.89, 1.68)	0.83 (0.48, 1.46)	1.16 (0.69, 1.93)
Q5	0.89 (0.60, 1.32)	1.00 (0.67, 1.51)	1.16 (0.83, 1.62)	1.01 (0.56, 1.80)	0.78 (0.44, 1.38)
*P-value*	0.071	0.796	0.162	0.731	0.630

^1^ Components of the metabolic syndrome were defined as (1) abdominal adiposity (waist circumference > 88 cm for women and > 102 for men); (2) low HDL levels (< 50 mg/dl); (3) high triglyceride levels (> 150 mg/dl); (4) high blood pressure (> 130/85 mmHg); and (5) high glucose levels (fasting plasma glucose > 100 mg/dl)

^2^ Data are reported as odds ratio (95% CI)

^3^ Adjusted for age, sex, energy intake, BMI, physical activity, education level, marital status, and smoking

Table 4 shows the odds ratio of metabolic syndrome according to quintiles of dietary patterns. There was no significant association between the healthy dietary pattern and the odds ratio of metabolic syndrome in the crude and adjusted model 1. However, in the fully adjusted model for age, sex, energy intake, BMI, physical activity, education level, marital status, and smoking, the odds ratio of metabolic syndrome was significantly decreased by 46% in subjects at the highest quintile of the healthy dietary pattern compared to those at the lowest quintile (OR = 0.54, 95% CI: 0.35, 0.84, P trend = 0.032). For the western dietary pattern, although an inverse association was observed in the fourth and fifth quintiles in the crude analysis, this dietary pattern was not significantly associated with the odds ratio of metabolic syndrome after adjusting for age, sex, and energy intake, as well as in the fully adjusted model. For the traditional dietary pattern, a considerable association with metabolic syndrome was observed neither in crude nor in adjusted models.

**Table 4:** The odds ratio of metabolic syndrome according to quintiles of dietary patterns^1^

	Healthy Dietary Pattern			Western Dietary Pattern			Traditional Dietary pattern		
	Crude	Model 1^2^	Model 2^3^	Crude	Model 1^2^	Model 2^3^	Crude	Model 1^2^	Model 2^3^
Q1	1	1	1	1	1	1	1	1	1
Q2	0.85 (0.64, 1.13)	0.81 (0.61, 1.09)	0.67 (0.46, 0.97)	1.03 (0.79, 1.35)	1.20 (0.90, 1.60)	1.38 (0.99, 1.92)	1.17 (0.87, 1.56)	1.05 (0.78, 1.41)	1.03 (0.71, 1.49)
Q3	0.81 (0.61, 1.08)	0.73 (0.54, 0.99)	0.65 (0.44, 0.94)	0.76 (0.57, 1.01)	0.92 (0.67, 1.25)	1.03 (0.71, 1.49)	1.20 (0.89, 1.60)	1.03 (0.77, 1.39)	1.05 (0.72, 1.52)
Q4	1.00 (0.75, 1.31)	0.87 (0.64, 1.19)	0.75 (0.51, 1.10)	0.62 (0.46, 0.83)	0.86 (0.60, 1.21)	1.12 (0.73, 1.72)	1.33 (1.00, 1.77)	1.09 (0.81, 1.48)	1.01 (0.69, 1.48)
Q5	0.84 (0.64, 1.12)	0.74 (0.53, 1.04)	0.54 (0.35, 0.84)	0.71 (0.53, 0.94)	1.16 (0.78, 1.71)	1.41 (0.84, 2.36)	1.11 (0.82, 1.48)	0.96 (0.70, 1.31)	0.96 (0.65, 1.44)
*P-trend*	0.599	0.196	0.032	< 0.001	0.793	0.542	0.303	0.926	0.857

^1^ Data are reported as odds ratio (95% CI)

^2^ Adjusted for age, sex, and energy intake

^3^ Adjusted for age, sex, energy intake, BMI, physical activity, education level, marital status, and smoking

## Discussion

This cross-sectional study was the first to examine the association between major dietary patterns and metabolic syndrome in a large sample of Iranian subjects in the coastal southern area. We identified three major dietary patterns (healthy, western, and traditional) among this population. We observed that the healthy dietary pattern characterized by a high consumption of vegetables, fruits, nuts, olives, and dairy products was associated with a 46% reduction in metabolic syndrome. This healthy pattern also showed an inverse association with abnormal individual metabolic syndrome components such as blood glucose, waist circumference, and blood pressure. The present findings are generally consistent with several previous research suggesting a healthy dietary pattern (rich in fruits, vegetables, nuts, dairy, and legumes) protects against metabolic syndrome [16–18], however, there is also evidence that has not found an association between healthy/prudent dietary patterns and metabolic syndrome [19–22].

Our participants with higher adherence to the healthy dietary pattern had higher intakes of fruits, vegetables, legumes, nuts, dairy products, fiber, magnesium, vitamin C, B6, and B9 and lower intakes of saturated fat, refined grains, and processed meats which can be attributed to the effectiveness of this pattern in improving metabolic syndrome components. The independent protective effects of low intake of food items such as refined grain [23] and saturated fatty acid (from processed meats) [24], and high intake of complex carbohydrates [23], fruits and vegetables [25], nuts [26], and legumes [27] has been strongly supported by evidence. It has been suggested that fruits, vegetables, and legumes which are rich in magnesium, fiber, and vitamin C play roles in reducing the risk of metabolic syndrome [28–30]. The dairy products high in calcium in a healthy dietary pattern, is also supposed to be effective in reducing abdominal obesity and lowering blood pressure [31, 32]. Moreover, a healthy diet with a low glycemic load can be associated with a reduced risk of insulin resistance as a key mediator of metabolic syndrome [33]. In general, the protective effect of plant-based dietary indices has been shown on metabolic syndrome [34]. There is a strong body of evidence to support that adherence to different types of healthy dietary patterns which contain high amounts of fruits, vegetables, whole grains, dairy products, nuts, and legumes could be effective in improving the components of metabolic syndrome in particular blood glucose control and blood pressure [35–40].

Our data did not show a different chance of metabolic syndrome according to quintiles of adherence to the western dietary pattern. This finding is in line with several studies that did not find a significant association between the western dietary pattern and metabolic syndrome and its components [17, 41–43]. The main food items in the western dietary pattern such as red meats, processed meats, proteins, fats, and saturated fats have resulted in a reduction of carbohydrate and sugar intakes in this pattern [41]. It is consequently possible that it has caused no significant inverse association of the western dietary pattern with metabolic syndrome. Our results also showed that the traditional dietary pattern with high content of sugar, tea, salt, potatoes, hydrogenated fats, and coffee were not associated with metabolic syndrome. This pattern is similar to the dietary pattern of high fat, sweets, and coffee found in the Kim et al. study among South Korean adults, which also indicated no significant correlation with metabolic syndrome [44].

We must point out the limitation of this study which was its cross-sectional design, because since these type of studies are helpful in assessing individuals’ dietary patterns, they cannot establish a causal and temporal relationship between dietary patterns and health outcomes. Moreover, the dietary intakes were assessed by using a semi-quantitative FFQ, which is prone to measurement error. The non-completion of the Iranian food composition table of Iran made us use the US Department of Agriculture (USDA) data bank in this study. The findings may also not be generalizable or produce an accurate description of all populations, however, we tried to consider many potential confounders in the data analysis.

## Conclusions

This cross-sectional study on Iranian adults in the coastal southern area revealed that a healthy dietary pattern is associated with a reduced risk of metabolic syndrome and its components, such as elevated waist circumference, high blood pressure, and high fasting blood sugar. It therefore seems that adherence to a healthy dietary pattern that includes fruits, vegetables, nuts, dairy, and legumes can be recommended in improving metabolic factors related to metabolic syndrome as a promising lifestyle strategy. Longitudinal studies are still needed to confirm our results in different populations.

### Electronic supplementary material

Below is the link to the electronic supplementary material.

Supplementary Material 1

## Data Availability

The datasets used and/or analyzed during the current study are available from the corresponding author upon reasonable request.

## Abbreviations

BKNCD: Bandare-Kong Non-Communicable Diseases
BMI: Body mass index
BP: Blood pressure
CI: Confidence interval
FFQ: Food frequency questionnaire
HC: Hip circumference
HDL-C: High-density lipoprotein cholesterol
LDL-C: Low-density lipoprotein cholesterol
MET: Metabolic equivalent
NCEP-ATP III: National Cholesterol Education Program Adult Treatment Panel III
OR: Odds ratio
PERSIAN: Prospective Epidemiological Research Studies in Iran
SD: Standard deviations
WC: Waist circumference

## References

[1] Bovolini A, Garcia J, Andrade MA, Duarte JA (2021). Metabolic syndrome pathophysiology and predisposing factors. Int J Sports Med.

[2] Swarup S, Goyal A, Grigorova Y, Zeltser R (2022). Metabolic syndrome.

[3] Santulli G. Dietary components and metabolic dysfunction: translating preclinical studies into clinical practice. MDPI; 2016. p. 632. 10.3390/nu8100632 PMC5084019 27754375

[4] Castro-Barquero S, Ruiz-León AM, Sierra-Pérez M, Estruch R, Casas R (2020). Dietary strategies for metabolic syndrome: a comprehensive review. Nutrients.

[5] Chauhan H, Belski R, Bryant E, Cooke M. Dietary Assessment Tools and Metabolic Syndrome: Is It Time to Change the Focus? Nutrients. 2022;14(8):1557. 10.3390/nu14081557 PMC9032662 35458121

[6] Kirti K, Singh SK (2023). Obesogenic diet and metabolic syndrome among adolescents in India: data-driven cluster analysis. BMC Cardiovasc Disord.

[7] Fabiani R, Naldini G, Chiavarini M. Dietary patterns and metabolic syndrome in adult subjects: a systematic review and Meta-analysis. Nutrients. 2019;11(9). 10.3390/nu11092056 PMC6770202 31480732

[8] McGuire S. Scientific Report of the 2015 Dietary Guidelines Advisory Committee. Washington, DC: US Departments of Agriculture and Health and Human Services, 2015. Adv Nutr. 2016;7(1):202-4. 10.3945/an.115.011684 PMC4717899 26773024

[9] Zoghi G, Kheirandish M (2021). Prevalence of type 2 diabetes, obesity, Central Obesity, and metabolic syndrome in a South Coastal Region, Iran, the PERSIAN Bandare Kong Cohort Study: a brief report. Hormozgan Med J.

[10] Poustchi H, Eghtesad S, Kamangar F, Etemadi A, Keshtkar AA, Hekmatdoost A (2018). Prospective Epidemiological Research Studies in Iran (the PERSIAN Cohort Study): Rationale, objectives, and design. Am J Epidemiol.

[11] Nejatizadeh A, Eftekhar E, Shekari M, Farshidi H, Davoodi SH, Shahmoradi M (2022). Cohort profile: Bandar Kong prospective study of chronic non-communicable diseases. PLoS ONE.

[12] Aadahl M, Jorgensen T (2003). Validation of a new self-report instrument for measuring physical activity. Med Sci Sports Exerc.

[13] Ainsworth BE, Haskell WL, Whitt MC, Irwin ML, Swartz AM, Strath SJ (2000). Compendium of physical activities: an update of activity codes and MET intensities. Med Sci Sports Exerc.

[14] Dare S, Mackay DF, Pell JP (2015). Relationship between smoking and obesity: a cross-sectional study of 499,504 middle-aged adults in the UK general population. PLoS ONE.

[15] Eghtesad S, Hekmatdoost A, Faramarzi E, Homayounfar R, Sharafkhah M, Hakimi H et al. Validity and reproducibility of a food frequency questionnaire assessing food group intake in the PERSIAN Cohort Study. Front Nutr. 2023;10. 10.3389/fnut.2023.1059870 PMC10436288 37599697

[16] Tamura Y, Omura T, Toyoshima K, Araki A (2020). Nutrition management in older adults with diabetes: a review on the importance of shifting prevention strategies from metabolic syndrome to frailty. Nutrients.

[17] Fabiani R, Naldini G, Chiavarini M (2019). Dietary patterns and metabolic syndrome in adult subjects: a systematic review and meta-analysis. Nutrients.

[18] Li T, Tang X, Liu Y, Li Y, He B (2021). Dietary patterns and metabolic syndrome among urbanized tibetans: a cross-sectional study. Environ Res.

[19] Arisawa K, Uemura H, Yamaguchi M, Nakamoto M, Hiyoshi M, Sawachika F (2014). Associations of dietary patterns with metabolic syndrome and insulin resistance: a cross-sectional study in a Japanese population. J Med Invest.

[20] Denova-Gutierrez E, Castanon S, Talavera JO, Gallegos-Carrillo K, Flores M, Dosamantes-Carrasco D (2010). Dietary patterns are associated with metabolic syndrome in an urban Mexican population. J Nutr.

[21] Akter S, Nanri A, Pham NM, Kurotani K, Mizoue T (2013). Dietary patterns and metabolic syndrome in a Japanese working population. Nutr Metab (Lond).

[22] Lutsey PL, Steffen LM, Stevens J (2008). Dietary intake and the development of the metabolic syndrome: the atherosclerosis risk in communities study. Circulation.

[23] Guo H, Ding J, Liang J, Zhang Y (2021). Associations of Whole Grain and Refined Grain Consumption with metabolic syndrome. A Meta-analysis of Observational studies. Front Nutr.

[24] Kim Y, Je Y. Meat consumption and risk of metabolic syndrome: results from the Korean Population and a Meta-analysis of Observational studies. Nutrients. 2018;10(4). 10.3390/nu10040390 PMC5946175 29565803

[25] Nguyen HD, Oh H, Kim M-S (2022). Higher intakes of fruits, vegetables, and multiple individual nutrients is associated with a lower risk of metabolic syndrome among adults with comorbidities. Nutr Res.

[26] Hassannejad R, Mohammadifard N, Kazemi I, Mansourian M, Sadeghi M, Roohafza H (2019). Long-term nuts intake and metabolic syndrome: a 13-year longitudinal population-based study. Clin Nutr.

[27] Martín-Cabrejas M. Legumes: an overview. Legumes: Nutritional Quality, Processing and Potential Health Benefits. 2019:1–18.

[28] Piuri G, Zocchi M, Della Porta M, Ficara V, Manoni M, Zuccotti GV (2021). Magnesium in obesity, metabolic syndrome, and type 2 diabetes. Nutrients.

[29] Wei B, Liu Y, Lin X, Fang Y, Cui J, Wan J (2018). Dietary fiber intake and risk of metabolic syndrome: a meta-analysis of observational studies. Clin Nutr.

[30] Guo H, Ding J, Liu Q, Li Y, Liang J, Zhang Y (2021). Vitamin C and metabolic syndrome: a meta-analysis of observational studies. Front Nutr.

[31] Cormick G, Belizán JM (2019). Calcium intake and health. Nutrients.

[32] Bhavadharini B, Dehghan M, Mente A, Rangarajan S, Sheridan P, Mohan V (2020). Association of dairy consumption with metabolic syndrome, hypertension and diabetes in 147 812 individuals from 21 countries. BMJ Open Diabetes Res Care.

[33] Elyasi L, Borazjani F, Ahmadi Angali K, Hosseini SA, Saki N (2024). Dietary insulin index, dietary insulin load and dietary patterns and the risk of metabolic syndrome in Hoveyzeh Cohort Study. Sci Rep.

[34] Lanuza F, Meroño T, Zamora-Ros R, Bondonno NP, Rostgaard-Hansen AL, Sánchez-Pla A (2023). Plasma metabolomic profiles of plant-based dietary indices reveal potential pathways for metabolic syndrome associations. Atherosclerosis.

[35] Ghaedi E, Mohammadi M, Mohammadi H, Ramezani-Jolfaie N, Malekzadeh J, Hosseinzadeh M (2019). Effects of a paleolithic Diet on Cardiovascular Disease Risk factors: a systematic review and Meta-analysis of Randomized controlled trials. Adv Nutr.

[36] Kastorini CM, Milionis HJ, Esposito K, Giugliano D, Goudevenos JA, Panagiotakos DB (2011). The effect of Mediterranean diet on metabolic syndrome and its components: a meta-analysis of 50 studies and 534,906 individuals. J Am Coll Cardiol.

[37] Ndanuko RN, Tapsell LC, Charlton KE, Neale EP, Batterham MJ (2016). Dietary patterns and blood pressure in adults: a systematic review and Meta-analysis of Randomized controlled trials. Adv Nutr.

[38] Ramezani-Jolfaie N, Mohammadi M, Salehi-Abargouei A (2019). The effect of healthy nordic diet on cardio-metabolic markers: a systematic review and meta-analysis of randomized controlled clinical trials. Eur J Nutr.

[39] Saneei P, Salehi-Abargouei A, Esmaillzadeh A, Azadbakht L (2014). Influence of Dietary approaches to stop hypertension (DASH) diet on blood pressure: a systematic review and meta-analysis on randomized controlled trials. Nutr Metab Cardiovasc Dis.

[40] Zimorovat A, Mohammadi M, Ramezani-Jolfaie N, Salehi-Abargouei A (2020). The healthy Nordic diet for blood glucose control: a systematic review and meta-analysis of randomized controlled clinical trials. Acta Diabetol.

[41] Hassannejad R, Kazemi I, Sadeghi M, Mohammadifard N, Roohafza H, Sarrafzadegan N (2018). Longitudinal association of metabolic syndrome and dietary patterns: a 13-year prospective population-based cohort study. Nutr Metab Cardiovasc Dis.

[42] Naja F, Shivappa N, Nasreddine L, Kharroubi S, Itani L, Hwalla N (2017). Role of inflammation in the association between the western dietary pattern and metabolic syndrome among Lebanese adults. Int J Food Sci Nutr.

[43] Choi JH, Woo HD, Lee JH, Kim J (2015). Dietary patterns and risk for metabolic syndrome in Korean women: a cross-sectional study. Med (Baltim).

[44] Kim J, Jo I (2011). Grains, vegetables, and fish dietary pattern is inversely associated with the risk of metabolic syndrome in South Korean adults. J Am Diet Assoc.

