# Supplementary material for: The association between dietary patterns and metabolic syndrome among Iranian adults, a cross-sectional population-based study (findings from Bandare-Kong non-communicable disease cohort study)
Source: BMC Endocr Disord. 2024 Apr 30;24:57. doi: 10.1186/s12902-024-01584-7 (PMC11059651; doi:10.1186/s12902-024-01584-7)
Supplement: Supplementary file 1 — Supplementary Material 1 [file 12902_2024_1584_MOESM1_ESM.docx]

**Supplementary Table 1-** Food grouping used for factor analysis

| Food groups | Food items |
| --- | --- |
| Red meats | Red meat, Kebab. Hamburgers |
| Processed meats | Sausages |
| Organ meats | Beef liver, Tongue, Brain, Lamb organ (Tripe, Head and trotters, Foot, Abomasum) |
| Fish | Fish, Salted fish, Shrimp/Crab, Bandar Kong fish |
| Canned fish | Canned fish |
| Poultry | Chicken, Other parts of chicken (Liver, Heart, Gizzard) |
| Eggs | Eggs |
| Hydrogenated fats | Solid oil, palm oil |
| Butter | Butter |
| Margarine | Margarine |
| Dairy products | Milk, Yogurt |
| High fat dairy products | Cheese, Ice-cream, Flavored milk. Chocolate milk. Coffee milk. Honey milk, Cream, Curd |
| Fruits | Pears, Apricots, Cherries, Sour cherry, Apples, Grapes, Bananas, Cantaloupe, Melons, Watermelon, Kiwi, Strawberries, Peaches, Mulberry, Plums, Persimmons, Pomegranates, Figs, Dates, Greengage, Jaam, Mango, Konar, Citrus fruits, All types of canned fruits, All types of natural fruit juices |
| Cruciferous vegetables | Cabbage. White Cabbage. Red Cabbage. Broccoli |
| Yellow vegetables | Carrots |
| Dried fruits | Dried Fig. Dried plum. Dried apricot. Dried peach, Dried mulberry. Raisin |
| Tomatoes | Tomatoes, Tomato paste |
| Vegetables | Cucumber, Vegetables, Cooked vegetables, Eggplant, Squash, Celery. Cooked celery, Green peas, Onion, Beetroot. Turnip, Ripe mushrooms, Corn, Sweet pepper, Green pepper, Green beans, Garlic |
| Green leafy vegetables | Chopped lettuce |
| Legumes | Mung bean. Lentil, Beans, Soy/Soy bean, Cotyledon, Peas, Cotyledon, Broad beans |
| Potatoes | Potatoes. French fries |
| Whole grains | Barbari and Taftoon breads, Sangak bread, Cooked barely and Bulgur, Barley bread, Diet bread, Kakel and Chamchamoo breads |
| Refined grains | Lavash bread, Baguette bread, Cooked pasta, Cooked rice |
| Pizza | Pizza |
| Snacks | Potato chips, Corn puffs, Biscuit wafer |
| Nuts | Walnut, Peanut, Other nuts (Almond. Almond Hindi. Pistachio. Hazelnut), Squash, Sunflower and Watermelon seeds |
| Mayonnaise | Mayonnaise sauce |
| Olive | Olives, Olive oil |
| Vegetable oils | Vegetable oils (except for olive oil) |
| Sugars | Sugar, Sugarloaf |
| Sweets and desserts | Chocolates, Dry cakes and sweets, Creamy cakes and sweets, Types of halva, Palm sap, Honey, Jam, Other sweets |
| Condiments | Local spices, Industrial lemon juice, tamarind, Salted fish juice, Mahyave. Soragh, Other pastes, Nido milk |
| Soft drinks | Soft drinks, Non-alcoholic beer |
| Yoghurt drink | Doogh |
| Salt | Salt |
| Pickles | Pickles, Pickled cucumber, Salinity |
| Coffee | Coffee |
| Tea | Tea |

**Supplementary Table 2-** Factor loading for food groups based on the major dietary pattern extracted from principal component analysis

| Food groups | Dietary patterns | | |
| --- | --- | --- | --- |
|  | **Healthy** | **Western** | **Traditional** |
| Vegetables | .664 |  |  |
| Fruits | .658 |  |  |
| Yellow vegetables | .572 |  |  |
| Green leafy vegetables | .560 |  |  |
| Cruciferous vegetables | .531 |  |  |
| Nuts | .483 |  |  |
| Tomatoes | .478 |  |  |
| Dried fruits | .477 |  |  |
| Olive | .407 |  |  |
| Dairy products | .335 |  |  |
| Pickles | .281 | .253 |  |
| Yoghurt drink | .259 |  |  |
| Legumes | .241 | .234 |  |
| Butter | .203 |  |  |
| Fish | .202 |  |  |
| Margarine |  |  |  |
| Vegetable oils |  |  |  |
| Soft drinks |  | .521 |  |
| Sweets and desserts |  | .496 |  |
| Condiments |  | .472 |  |
| Pizza |  | .455 | -.201 |
| Red meats | .206 | .449 |  |
| Snacks |  | .446 |  |
| Poultry |  | .441 |  |
| Refined grain |  | .390 |  |
| Mayonnaise |  | .378 |  |
| Canned fish |  | .345 |  |
| Eggs |  | .338 |  |
| Processed meats |  | .320 |  |
| High fat dairy products |  | .317 |  |
| Organ meats |  | .278 |  |
| Whole grains | .206 | .220 |  |
| Sugars |  |  | .825 |
| Tea |  |  | .816 |
| Salt |  |  | .254 |
| Potatoes |  | .237 | .242 |
| Hydrogenated fats |  |  | .233 |
| Coffee |  |  | .223 |
| Percentage of variance explained (%) | **10.20** | **6.30** | **4.28** |

Factor loading less than 0.20 were excluded
